# Relationship Between the Duration of Hospitalization and Readmission Status in Patients With Schizophrenia: A Saudi Arabian Cohort Study

**DOI:** 10.1002/brb3.70807

**Published:** 2025-09-08

**Authors:** Mahmoud A. Alharthi, Rajaa M. Al‐Raddadi, Sulhi A. Alfakeh

**Affiliations:** ^1^ Public Health Senior Specialist, Population Health Management Jeddah First Health Cluster Riyadh Saudi Arabia; ^2^ Faculty of Medicine, Department of Community Medicine King Abdulaziz University Jeddah Saudi Arabia; ^3^ Faculty of Medicine, Department of Internal Medicine, Psychiatry Unit King Abdulaziz University Jeddah Saudi Arabia

**Keywords:** length of hospitalization, readmission, schizophrenia, mental health, Jeddah

## Abstract

**Introduction:**

The readmission of individuals with schizophrenia to inpatient care poses a significant challenge for health practitioners, as this tendency has a culminating effect on the patients and their families. The duration of hospitalization of a patient with schizophrenia in a hospital or mental health facility poses a significant burden on mental healthcare systems. This study aimed to assess the length of stay of patients with schizophrenia in a mental health facility. The relationship between the duration of hospitalization and risk of readmission for these patients was assessed with respect to the sociodemographic endpoints.

**Methods:**

This retrospective cohort study included 145 individuals who were admitted to the Eradah Mental Health Complex in Jeddah from July 1, 2018 to December 31, 2018.

**Results:**

The present study revealed that 84.8% of the sample comprised male participants. Moreover, only 5.5% of the patients were employed, with a larger proportion being unemployed. Among the total admitted patients, 36.6% were readmitted within a year, and the average length of stay was 49.1 days. Importantly, there was no discernible relationship between the readmission status and length of stay in the present study.

**Conclusion:**

The current study suggested that enhancing the transition from inpatient psychiatric care to outpatient care may result in positive disease outcomes, which may possess clinical implications for reducing the length of hospitalization of patients. This can culminate in reducing the burden of disease on the patients, their families, and ultimately, the healthcare system.

## Graphical abstract

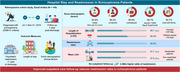

## 1 Introduction

Schizophrenia is a long‐term and severe mental health condition that involves distortions in various aspects of cognition, such as thinking, perception, emotions, language, self‐concept, and behavior. The primary symptoms of schizophrenia include hallucinations, which involve hearing voices or seeing things that are not present, and delusions, which involve fixed and false beliefs. Furthermore, the patient may experience influence, passivity, or control by external forces. The behavior of individuals diagnosed with schizophrenia may appear bizarre, purposeless, unpredictable, or accompanied by inappropriate emotional responses that can interfere with the organization of their behavior.

Schizophrenia is among the top 15 disorders worldwide, resulting in significant disabilities (El Morr et al. 2021). The impact of mental illness extends to individuals, families, healthcare systems, and economies. Furthermore, mental illness has attracted national attention owing to its associated social challenges; in particular, schizophrenia poses a significant national health challenge that can cause extensive damage at individual, social, and economic levels. Therefore, the implementation of ongoing healthcare strategies is crucial for preventing relapse following intensive care (Jung et al. 2021).

The prevalence of schizophrenia has risen from 13.1 million cases in 1990 to 20.9 million cases in 2016 on a global scale (Charlson et al. 2018). It affects approximately 1% of the population, with an incidence rate of nearly 1.5 cases per 10,000 individuals (McGrath et al. 2008). A previous study reported that 13% of patients treated in psychiatric outpatient facilities in Saudi Arabia had schizophrenia. Furthermore, this study revealed that half of those admitted to inpatient mental hospitals are likely to have schizophrenia (Almasabi 2013). Moreover, according to data reported by the Saudi Ministry of Health (MOH), 13,363 frequent and new cases were admitted to psychiatric inpatient departments; moreover, 145,625 frequent and new cases visited psychiatric outpatient clinics with diagnoses of schizophrenia, schizotypal disorders, and delusional disorders (Reis and Tsai 2022). These findings highlighted a significant public health concern.

Disability‐adjusted life years (DALYs) are the metrics used by the World Health Organization to assess the overall burden of disease; DALYs take into consideration both the years of life lost and years lived with a disability. This metric was applied to estimate the global burden of schizophrenia in 2017, which resulted in 1.13 million cases and 12.66 DALYs per 100,000 individuals (195.27 DALYs per 100,000 individuals globally) (Ji et al. 2018; Tiihonen 2011). In Saudi Arabia, the DALYs from schizophrenia were estimated at 124.89 per 100,000 individuals in 2017, which was lower than the global rate of 36% for the same year (Jung et al. 2021; Feigin et al. 2021).

A significant number of individuals diagnosed with schizophrenia tend to relapse following periods of improvement, remission, or even recovery (Ji et al. 2018). Relapse often leads to negative consequences, including a decline in both psychosocial and occupational functioning (Pillai et al. 2018). Patients diagnosed with schizophrenia frequently experience relapse even after the completion of treatment, and they may need readmission to the hospital several times within a few years following the initial onset of a psychotic episode (Ji et al. 2018; Tiihonen 2011). Readmission can have a profound impact on both the patients and their families, resulting in increased mental health care costs and disruptive experiences. This phenomenon has been described using various terms in the literature, including rehospitalization and recidivism, to emphasize the need for costly, frequent episodes of inpatient care (Lien 2002; Hewlett and Moran 2014).

One of the key global indicators commonly employed in healthcare to identify the occurrence of readmission is the readmission rate (Yu et al. 2015). As a quality assessment tool, this indicator has garnered the attention of health sector policy makers (Rumball‐Smith and Hider 2009). Moreover, readmission should be acknowledged as an indicator of either worsening of the patient's condition or relapse of illness (Jeppesen et al. 2016).

According to a study conducted by the Organization for Economic Cooperation and Development (OECD) in 2011 across 15 countries, a considerable proportion of patients diagnosed with schizophrenia were found to be readmitted after being discharged. An OECD study reported an overall unplanned readmission rate of 13% within 30 days among discharged patients with schizophrenia (Hewlett and Moran 2014). Additionally, another study estimated the 30‐day readmission rate to psychiatric beds to be 10.4% in six European countries (Katschnig et al. 2019). In Korea, in 2017, the readmission rate within 180 days for schizophrenia, schizotypal, and delusional disorders was estimated to be 46.8% (Jung et al. 2021). A similar study in Oman demonstrated a readmission rate of 39% for readmitted patients within 1 year of index admission (Al‐Shehhi et al. 2017). Furthermore, a study in Saudi Arabia estimated a readmission rate of 83.3% among patients diagnosed with schizophrenia in four central psychiatric hospitals overseen by the MOH, thus highlighting readmission as a significant public health concern for this population (Parentela et al. 2019).

Numerous factors associated with psychiatric readmission are not defined solely by the severity of the mental illness (Roick et al. 2004); one such factor is the duration of stay, which has been explored in several studies that found a correlation between a shorter stay and an increased readmission rate (Lin et al. 2006; Boaz et al. 2013). However, the impact of the length of stay varies across European countries. For instance, in Finland and Norway, a longer stay was associated with a decrease in the psychiatric readmission rates, while the opposite was true in Romania (Katschnig et al. 2019).

Our study aimed to investigate the association between the length of hospitalization (LOH) and the likelihood of readmission among individuals diagnosed with schizophrenia at the Eradah Mental Health Complex in Jeddah, Saudi Arabia. The primary objective of our study was to determine the LOH for patients with schizophrenia and the risk of readmission for these patients.

## 2 Materials and Methods

### 2.1 Study Design

This study was conducted using a retrospective cohort design and involved an analysis of the medical records. This study adhered to the guidelines outlined in the Strengthening the Reporting of Observational Studies in Epidemiology (STROBE) statement.

### 2.2 Target Study Population

The target study population included patients diagnosed with schizophrenia and subsequently admitted to Eradah Mental Health Complex. Specifically, their index admission, which served as the baseline and first admission, occurred between July 1, 2018, and December 31, 2018. Following their discharge, the patients were monitored for a period of 1 year, with the observation period ending on the same date as their index admission (for instance, if the patient's index admission was on July 1, their observation period would conclude on June 30, 2019).

### 2.3 Study Setting

The study was conducted at the Eradah and Mental Health Complex, which is situated at the south of Jeddah and boasts a capacity of 125 beds. The hospital offers a comprehensive range of services, including psychiatric outpatient care, inpatient treatment, home care, and emergency care.

### 2.4 Inclusion Criteria

The study's inclusion criteria were patients who met the Diagnostic and Statistical Manual of Mental Disorders (DSM‐IV) criteria for schizophrenia, were of either sex, had a primary diagnosis of schizophrenia, were clinically stable, were at least 18 years old, and were hospitalized in acute psychiatric wards from July 1 to December 31.

### 2.5 Exclusion Criteria

Exclusion criteria included patients who were discharged owing to physical illness, death, or legal issues, or if the diagnosis was converted to a diagnosis other than schizophrenia.

### 2.6 Sample Size

We included all the patients who satisfied the inclusion criteria and were admitted to the Eradah and Mental Health Complex with a diagnosis of schizophrenia between July 1, 2018, and December 31, 2018.

### 2.7 Data Collection

Our primary source of sociodemographic and clinical data was patients’ medical records. This record provided information on the patients’ sex, age, educational level, marital status, and living conditions. Additionally, we considered the patients’ employment status and income, as well as whether they received a social security salary and had evidence of poor social support. Considering the clinical data, we recorded the total number of previous admissions and the type of index admission, as well as whether the patient was admitted voluntarily or involuntarily.

In our analysis, we considered factors such as aggression or self‐harm risk, substance abuse, adherence to medication, comorbidities, use of long‐acting injectable antipsychotics, and type of index discharge. The data were collected during a specific period, from July 1, 2018 to December 31, 2018. Additionally, the data were initially collected in printed form and then input into the Epi Info program version 7, developed by the Centers for Disease Control and Prevention.

### 2.8 Primary Outcome and Variables

The primary goal of the current study was to assess the length of stay of patients with schizophrenia within 1 year. The length of stay was classified into the following groups: Group 1, 1–7 days; Group 2, 8–14 days; Group 3, 15–30 days; Group 4, > 30 days. Moreover, we analyzed various sociodemographic factors, including age, sex, marital status, employment status, educational level, living conditions, social support, and income. Furthermore, we examined the clinical factors, such as previous hospitalizations, illness duration, type of index admission (voluntary or involuntary), substance abuse, treatment received at index admission, discharge type, use of long‐acting injectable antipsychotics, receipt of electroconvulsive therapy sessions, presence of comorbidities, treatment compliance, outpatient care follow‐up, and readmission.

### 2.9 Data Analysis

We used descriptive statistics, including frequencies and percentages, to summarize the sociodemographic characteristics and clinical profiles of the study participants. Continuous variables are expressed as mean and standard deviation (SD). The outcome of readmission among patients was assessed using the chi‐square test for categorical variables in a bivariate analysis. Additionally, we used Fisher's exact test when the assumptions of the chi‐square test were not met. An independent *t*‐test was used to analyze the continuous variables. Furthermore, we conducted multiple logistic regression analyses to adjust for confounding factors, identify potential readmission risk factors, and evaluate whether the bivariate variables could independently predict psychiatric readmission. A significance level of *p* < 0.05 was considered statistically significant. SPSS statistical software (Version 28, IBM) was used for the data analysis and all the analytical operations.

### 2.10 Ethical Approval

The ethical approval for this study was granted on April 27, 2020, by the Institutional Review Board (IRB) of the MOH (Registration number: H‐02‐J‐002). All the information for the study was obtained through a legal and cooperative process in compliance with the MOH guidelines. No hard copies of the participants’ data were maintained, and access to the data was limited to the investigator. The entered data were securely stored in a password‐protected drive on the researcher's computer, which was also protected by Internet security and antivirus software. Therefore, the data collection process did not pose any risk or harm to the rights of the study participants.

## 3 . Results

### 3.1 Sociodemographic Characteristics

The study included 145 adults diagnosed with schizophrenia who were hospitalized in inpatient wards at Eradah Mental Health Complex during the period from July 1, 2018 to December 31, 2018. As shown in Table 1, the majority of the participants were males (84.8%), whereas females comprised a minority at 15.2%. The mean age of all the patients was 34.2 years (±10.2), ranging from 18 to 64 years. Notably, the highest proportion of patients was within the age range of 18–29 years, with 37.2% of the study sample falling within this category.

**TABLE 1:** Descriptive sociodemographic data of the admitted patients (*n* = 145).

Variable		*n*	%
Sex	Male	123	84.8
	Female	22	15.2
Age (years)	18–29	54	37.2
	30–39	50	34.5
	≥ 40	41	28.3
Education level	Illiterate	8	5.5
	Less than high school	62	42.8
	High school	54	37.2
	Graduate	21	14.5
Marital status	Married	20	13.8
	Divorced and widowed	24	16.5
	Single	101	69.7
Living condition (*n* = 144)	Alone	20	13.9
	With family	122	84.7
	Institutionalized	2	1.4
Employment status	Employed	8	5.5
	Retired	11	7.6
	Unemployed	126	86.9
Income (Saudi Riyal) (*n *= 135)	< 3000	117	80.7
	3000–5000	9	6.2
	≥ 5000	9	6.2
Receiving social security salary (*n* = 138)	No	60	43.5
	Yes	78	56.5

Abbreviation: *n*, number.

In terms of educational attainment, the majority of patients in this study had not completed high school (48.3%). Additionally, the vast majority of the patients were unmarried, with 86.2% reporting being single. Furthermore, 84.7% of the patients lived with their families. According to the accommodation data, 27% of the patients resided outside Jeddah City, while only 5.5% were employed. A significant proportion of patients had an income of less than 3000 SR per month, with 80.7% falling into this category. The mean income for patients was 1501.1 SR (SD 2192.5 SR), and 43.5% of patients did not receive social security salaries.

### 3.2 Clinical Characteristics

In the sample of admitted patients with schizophrenia, the mean duration of illness was 9.7 years (SD: 7.14 years) and ranged from 1 to 38 years. Approximately 73.2% of their illness duration was ≥ 5 years, as depicted in Table 2. Furthermore, comorbidities were not present in 66.2% of the patients. Regarding medication compliance, 87.3% of the patients demonstrated noncompliance. All the patients received oral antipsychotic treatment, and 86.8% received long‐acting injectable antipsychotics, as shown in Table 2. Moreover, 77.2% of the patients did not undergo electroconvulsive therapy. In addition, 61.4% of the patients had no history of substance abuse. Furthermore, 63.4% of the patients posed no risk to others, and a significant proportion (92.4%) posed no risk to themselves. Additionally, 78.6% of the patients demonstrated no evidence of poor social support in their medical records.

**TABLE 2:** Descriptive data of the patient's clinical profile (*n* = 145).

Variable		*N*	%
Illness duration (years) (*n* = 142)	> 5 years	38	26.8
	5–10 years	54	38
	> 10 years	50	35.2
Presence of comorbidity	No	96	66.2
	Yes	49	33.8
Compliance to medication (*n *= 142)	No	124	87.3
	Yes	18	12.7
Receiving long‐acting injectable antipsychotic	No	19	13.1
	Yes	126	86.9
Receiving electroconvulsive therapy	No	112	77.2
	Yes	33	22.8
Substance abuse	No	89	61.4
	Yes	56	38.6
Aggression (risk to others)	No	92	63.4
	Yes	53	36.6
Risk of self‐harm	No	134	92.4
	Yes	11	7.6
Poor social support	No	114	78.6
	Yes	31	21.4

Abbreviation: *N*, number.

As shown in Table 3, the average duration of stay was 49.1 days, with a SD of 52.3. The number of days ranged from 3 to 323, with a median of 32 days. The largest proportion of patients belonged to Group 4 (52.4%), who had a longer stay of over 30 days in the inpatient wards. Furthermore, as shown in Table 4, the mean and median length of stay for readmitted patients were higher than those for the non‐readmitted patients.

**TABLE 3:** Descriptive data of the patient's length of stay (*n *= 145).

Variable		*n*	%
Length of stay groups	Group 1 (1–7 days)	4	2.8
	Group 2 (8–14 days)	24	16.6
	Group 3 (15–30 days)	41	28.3
	Group 4)> 30 days)	76	52.4

Abbreviation: *n*, number.

**TABLE 4:** Descriptive data of length of stay in terms of the readmission status (*n* = 145).

Variable		Non‐readmitted	Readmitted
Length of stay	Mean ± SD	47.6 ± 52.4	51.7 ± 52.5
	Median (IQR)	27 (41)	39 (41)

Abbreviations: IQR, interquartile range; SD, standard deviation.

**FIGURE 1:**
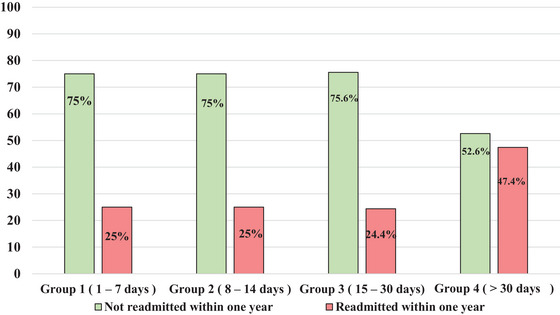
Occurrence of readmission within 1 year among the groups according to the length of stay.

Additionally, approximately 67.9% of the readmitted patients were categorized in Group 4, which corresponds to a length of stay exceeding 30 days. As depicted in Figure 1, nearly half of the patients in Group 4 were readmitted.

### 3.3 Risk Factors of Readmission

As shown in Table 5, no statistically significant variations were observed among the admitted patients based on factors such as sex, age, education, marital status, and living conditions. Similarly, these patients did not exhibit any notable disparities in employment status, income, or social security benefits.

**TABLE 5:** Bivariate analysis of the sociodemographic variables with the readmission status among the admitted patients (*n *= 145).

Variable		Readmission				*p*
		No		Yes		
		*n*	%	*n*	%	
Sex	Male	76	61.8	47	38.2	0.32^a^
	Female	16	72.7	6	27.3	
Age (years)	18–29	36	66.7	18	33.3	0.61^a^
	30–39	29	58	21	42	
	≥ 40	27	65.9	14	34.1	
Education level	Illiterate	3	37.5	5	62.5	0.16^b^
	Less than high school	38	61.3	24	38.7	
	High school	34	63	20	37	
	Graduate	17	81	4	19	
Marital status	Married	16	80	4	20	0.24^b^
	Divorced and widowed	15	62.5	9	37.5	
	Single	61	60.4	40	39.6	
Living condition (*n* = 144)	Alone	14	70	6	30	0.82^b^
	With family	76	62.3	46	37.7	
	Institutionalized	1	50	1	50	
Employment status	Employed	7	87	1	12.5	0.32^b^
	Retired	6	54.5	5	45.4	
	Unemployed	79	62.7	47	37.3	
Income (Saudi Riyal) (*n* = 135)	< 3000	71	60.7	46	39.9	0.73^b^
	3000–5000	6	66.7	3	33.3	
	≥ 5000	7	77.8	2	22.2	
Receiving social security salary (*n* = 138)	No	39	65	21	35	0.67^a^
	Yes	48	61.5	30	38.5	

Abbreviation: *n* = number.

^a^ *p* value conducted by a chi‐square test.

^b^ *p* value conducted by a Fisher‐exact test.

According to the clinical characteristics presented in Table 6, duration of illness, presence of comorbidities, and compliance with medication did not demonstrate a significant correlation with the occurrence of readmission. Additionally, no significant association was observed between readmission and long‐acting antipsychotic use. Electroconvulsive therapy did not have any significant effect. Moreover, substance abuse, risks posed to patients themselves or others, and poor social support did not show any significant differences.

**TABLE 6:** Bivariate analysis of the clinical variables with readmission among admitted patients (*n* = 145).

Variable		Readmission				*p*
		No		Yes		
		*n*	%	*n*	%	
Illness duration (Years) (*n* =142)	< 5 years	27	71.1	11	28.9	0.43^a^
	5–10 years	33	61.1	21	38.9	
	> 10 years	29	58	21	42	
Presence of comorbidity	No	64	66.7	32	33.3	0.26^a^
	Yes	28	57.1	21	42.9	
Compliance to medication (*n* = 142)	No	76	61.3	48	38.7	0.37^a^
	Yes	13	72.2	5	27.8	
Receiving long‐acting injectable antipsychotic	No	12	63.2	7	36.8	0.97^a^
	Yes	80	63.5	46	36.5	
Electroconvulsive therapy	No	72	64.3	40	35.7	0.70^a^
	Yes	20	60.6	13	39.4	
Substance abuse	No	61	68.5	28	31.5	0.10^a^
	Yes	31	55.4	25	45.6	
Aggression (Risk to others)	No	59	64.1	33	35.9	0.82^a^
	Yes	33	62.3	20	37.7	
Risk of self‐harm	No	87	64.9	47	35.1	0.21^a^
	Yes	5	45.5	6	54.5	
Poor social support	No	74	64.9	40	35.1	0.48^a^
	Yes	18	58.1	13	41.9	

^a^ *p* value conducted by a chi‐square test.

Compared to the number of previous admissions, notable variations were evident, as shown in Table 7 (*p < *0.04). Nearly 34% of the readmitted cases were among patients with more than three previous admissions. It is worth noting that there was no significant difference in the type of discharge from the index admission, with 62.5% of the unplanned discharged patients being readmitted.

**TABLE 7:** Bivariate analysis of the length of stay variable with the readmission status among the admitted patients (*n* = 145).

Variable		Readmission				*p*
		No		Yes		
		n	%	N	%	
Length of stay groups	Group one (1–7 days)	3	75	1	25	0.04* b
	Group two (8–14 days)	18	75	6	25	
	Group three (15–30 days)	31	75.6	10	24.4	
	Group four)< 30 days)	40	52.6	36	47.4	

*Significant, b, p value conducted by a Fisher‐exact test

Multiple logistic regression produced adjusted odds ratios, as shown in Tables 8 and 9. These ratios represent the relationship between one of the independent variables, namely the risk factors for readmission, and the dependent variable, readmission, after taking into account the other variables in the model. As shown in Table 8, a multiple logistic regression analysis was conducted to investigate the relationship between previous admissions, length of stay, follow‐up to outpatient care, and readmission. Furthermore, for follow‐up to outpatient care, the “yes” category was considered the reference group. The results indicated a statistically significant difference in the readmission rates between individuals who did not follow up with outpatient care and those who did. Specifically, the odds of readmission were 2.266 times higher in individuals who did not follow up with outpatient care compared to those who did, after adjusting for other variables in the model (Table 9). Logistic regression analysis demonstrated a significant influence of follow‐up to outpatient care on the risk of readmission.

**TABLE 8:** Crude odds ratio (95% confidence interval) from the logistic regression analysis identifying the association between the risk factors and readmission status.

Predictor variable	Crude OR	Confidence interval		*p* value
		Lower	Upper	
Previous admissions				
No previous admission (ref)				
One admission	0.758	0.269	2.133	0.599
Two admissions	1.240	0.365	4.206	0.730
Three admissions	1.278	0.433	3.770	0.656
More than three admissions	3.409	1.232	9.436	0.018^*^
Length of stay				
Group 1 (1–7 days) (ref)				
Group 2 (8–14 days)	1.000	0.087	11.525	1.000
Group 3 (15–30 days)	0.968	0.090	10.381	0.978
Group 4)> 30 days)	2.700	0.269	27.134	0.399
Follow‐up to outpatient care				
Yes (ref)				
No	3.098	1.534	6.257	0.002^*^
Length of stay				
Group 1 (1–7 days) (ref)				
Group 2 (8–14 days)	1.000	0.087	11.525	1.000
Group 3 (15–30 days)	0.968	0.090	10.381	0.978
Group 4)> 30 days)	2.700	0.269	27.134	0.399
Follow‐up to outpatient care				
Yes (ref)				
No	3.098	1.534	6.257	0.002^*^

Abbreviations: OR, odds ratio; Ref, reference category.

* significant.

**TABLE 9:** Adjusted odds ratio (95% confidence interval) from the logistic regression analysis identifying the association between risk factors and readmission status.

Predictor variable	Adjusted OR	Confidence interval		*p* value
		Lower	Upper	
Previous admissions				
No previous admission (ref)				
One admission	0.613	0.203	1.850	0.385
Two admissions	1.012	0.279	3.677	0.985
Three admissions	0.853	0.266	2.743	0.790
More than three admissions	2.082	0.686	6.315	0.195
Length of stay				
Group 1 (1–7 days) (ref)				
Group 2 (8–14 days)	0.973	0.077	12.341	0.983
Group 3 (15–30 days)	0.887	0.076	10.417	0.924
Group 4)>30 days)	2.177	0.196	24.164	0.527
Follow‐up to outpatient care				
Yes (ref)				
No	2.266	1.047	4.903	0.038^*^

* * Significant

## 4 Discussion

The results of this study regarding the patients’ sociodemographic characteristics did not vary from those of a previous local study. The similarities in our study with that by Parentela et al. were that the highest proportion of readmitted patients in our study were unmarried (92.5%), 73.6% were < 40 years old, with a mean age of 34.2 years. In addition, 90.2% of the patients had an income below 3000 SR, which is similar to that reported by Parentela et al. (2019) regarding the significant number of readmitted patients (80.1%) and their socioeconomic status in the low‐income category. However, because the study by Parental et al. was limited to male participants, we could not compare the studies in terms of sex. In addition, our study findings were consistent with those by Hung et al. (2017), which determined that younger age, with a mean of 35.9 years, unmarried patients, and male sex were predominant in the readmitted group.

A previous study found that male patients had a higher frequency of readmissions, which is consistent with our study findings (Gonçalves‐Pinho et al. 2021). However, the female patients in our study were less likely to be readmitted than those in the study by Pfiffner et al. (2014) Considering the educational level, we found similarities in the educational characteristics of the readmission group with Levine and Rabinowitz's study; the patients who did not finish school represented 54.7% in our study (Levine and Rabinowitz 2009). In contrast, Levine and Rabinowitz's study indicated that less education and early dropout from school among males were significantly associated with a risk of a greater number of admissions. However, no significant variations were observed in the essential demographic aspects between the readmission and non‐readmission groups, which was similar to the study by Chi et al. conducted in Taiwan in 2016 (Chi et al. 2016). Similarly, Krivoy et al. (2012) exhibited no differences in the patient demographic characteristics or readmission rate.

Regarding the clinical factors, considering the type of hospital admission, whether voluntary or involuntary (compulsory) index admission, and evidence of poor social support, our study found that patients who were admitted compulsorily or with poor social support were less frequent in the readmitted group and were statistically insignificant, which is different from the results of the previous studies regarding social support (van der Post et al. 2014; Sfetcu et al. 2017). The results showed that involuntary admission was not associated with a high risk of 1‐year readmission, which is inconsistent with the findings reported by Hung et al. (2017). Although Bulgari et al. (2017) and Zhang et al. (2011) found that physically aggressive patients with a risk to others at the time of being admitted (index admission) showed more involuntary admissions than non‐aggressive patients and an increased risk of readmission, with a statistically significant association, our study did not demonstrate a significant association. In contrast, the total hospital admissions before the index admission date had no significant association with readmission, which contradicts the study findings reported by Tulloch et al. (2016).

Furthermore, our findings demonstrated no risk of readmission when considering self‐harm among patients discharged from inpatient psychiatric wards, which varies from that reported by Gunnell et al. (2008). In addition, no significant impact was observed on readmission in the current study, considering the presence of comorbidities and substance abuse, which is contrary to the findings reported by Busch et al. (2015). Although previous studies have reported poor compliance with the treatment with the risk of relapse and readmission (Marcus et al. 2015; Adebiyi et al. 2018), our results demonstrated no statistical significance.

Moreover, no statistically significant relationship was observed between the use of long‐acting injectable antipsychotics and readmission in the current study. These results were consistent with those of Chou et al. (2016). Although the analysis by Chou et al. did not show any statistical significance, the use of long‐acting injectable antipsychotics decreased the number of readmissions when compared with the use of oral antipsychotics. These limitations may be attributed to the limited sample size (Chou et al. 2016). However, the use of long‐acting injectable antipsychotics is reportedly related to patient adherence to treatment and is a predictor of psychiatric readmission (Marcus et al. 2015; MacEwan et al. 2016).

In addition to the above, no association was observed between receiving electroconvulsive therapy sessions at discharge and the 1‐year readmission in the current study. However, electroconvulsive therapy during index admissions has been previously reported as a predictor of the readmission status (Zhang et al. 2011). Moreover, regarding the index discharge type, no association was observed with the 1‐year readmission rate in the current study. However, discharge planning and the type of index discharge (whether against medical advice or not) have been indicated in previous research as a risk and predictor of the readmission rate (Valevski et al. 2012). Our findings also revealed no significant association between the illness duration and readmission in the study population. In contrast, Adebiyi et al. (2018) reported an association between the illness duration and readmission rate.

As mentioned in previous studies based on LOH, the mean LOH for admitted patients was 49.1 days, which is higher than the mean length of stay reported by the Saudi MOH for convalescent and mental health hospitals in Saudi Arabia (29 days) (Chou et al. 2016). The group with a length of stay > 30 days constituted two‐thirds of the patients readmitted within 1 year, which may be attributed to the deterioration in the patients' abilities to perform activities of daily living independently, as reported by Chen et al. (2017). In addition, the severity of the psychiatric symptoms and deterioration in the ability to perform social and occupational functions, as reported by Bian et al., may explain this finding for the current study (Boyer et al. 2000). However, it is difficult to ascertain this because we were unable to measure the performance of these activities and functions in the patients in the current study.

Previous studies indicated that the readmission rate of patients diagnosed with schizophrenia was approximately 33.3%–86% after a 1–2‐year follow‐up (Parentela et al. 2019; Hung et al. 2017; MacEwan et al. 2016). In our study, the readmission rate within 1 year among patients with schizophrenia was 36.6%, which is within the range of the previously reported rates.

Finally, the multiple logistic regression analysis findings contradict those of previous studies (Lin et al. 2006; Hung et al. 2017; Vasudeva et al. 2009; Pono 2009; Dey et al. 2016). Despite the absence of statistical significance for the length of stay, the statistical analysis of the research recognized an association between follow‐up to outpatient care and readmission within 1 year. According to the systematic review by Sfetcu et al. (2017), this risk factor is considered a predictor of psychiatric readmission. Moreover, the results were similar to those by Nelson et al. (2000) regarding a significant association between outpatient care follow‐up within 1 year and the 365‐day readmission rate/

### 4.1 Study Strengths

The results of the current study are indispensable in various ways. First, this was a cohort study representing the LOH and rate of readmission for patients with schizophrenia in the Kingdom of Saudi Arabia. This study was conducted in the inpatient department of a psychiatric clinic in the city of Jeddah, Kingdom of Saudi Arabia. The results presented here can serve as the basis for researchers working in the fields of mental healthcare and psychiatric services. It is generally believed that wealthy Gulf countries are devoid of complex mental issues. This study examines mental health care and psychiatric services in Saudi Arabia, including the practice of psychiatric inpatient care, outpatient follow‐up, and upcoming research for educational purposes.

Furthermore, the results elucidate a well‐planned method to improve the quality of life of patients with schizophrenia, thus providing them with more accessibility to improved medical services.

### 4.2 Study Limitations

Although the study findings are valuable to Gulf countries, they still has some limitations that need to be discussed.

First, the data considered in this study are retrospective, lacking sociodemographic information and certain details regarding previous as well as index admissions. Second, the entire study was limited to one mental facility; thus, many facilities should be lined up for obtaining better and multifaceted results. Third, the sample size was not calculated owing to the absence of diagnoses of patients and visitors to the hospital for all the included study participants.

### 4.3 Recommendations

Improving the transition from psychiatric inpatient to outpatient care may help decrease the risk of subsequent hospital readmission in these patients. Moreover, healthcare providers should find suitable solutions to support caregivers and effectively transition them to outpatient care. In fact, relatively simple interventions may improve outpatient follow‐up care, including providing telephone case management, shortening the period between discharge and the first scheduled follow‐up appointment as much as possible, and arranging appointments preceding discharge with the outpatient care clinician as soon as possible (Boyer et al. 2000, Kolbasovsky et al. 2010). In addition, arranging outpatient appointments is an effective planning practice and should be considered an essential goal in psychiatric clinical settings as an improvement and quality measurement tool. Furthermore, the engagement of family members, proactive discharge planning, and shared decision‐making may also be essential to ensure continuity of care following hospital discharge. Hopefully, these recommendations will contribute to lower psychiatric readmission rates.

## 5 Conclusions

Our study demonstrated that the LOH can be considered a predictive factor for disease burden. This correlation was positive when the LOH was higher, and the disease burden was also alleviated. Moreover, our results showed that interruptions in healthcare service delivery during the transition from the hospital to psychiatric outpatient care were common among patients with schizophrenia. Furthermore, the likelihood of readmission within 1 year following inpatient discharge was directly associated with follow‐up to outpatient care, which was the only significant predictor.

Considering the expansion of the current study, future researchers should include mental health and psychiatric hospitals in Saudi Arabia to obtain generalizable findings specific to this population. The main mental health hospitals are distributed across different central regions of Saudi Arabia. These hospitals should coordinate their research, development, and improvement projects for inpatient and outpatient services to achieve shared goals.

In addition to the recommendation above, researchers should utilize longitudinal and prospective designs in the future to determine the risk factors that contribute to relapse and readmission. Furthermore, utilizing this cohort design in follow‐up patients over a long period of time is advantageous in studying the occurrence of readmission for a specific exposure variable change over time while eliminating recall bias. In addition, researchers will be able to determine the readmission rates based on the location, healthcare setting, and specific populations.

## Author Contributions

**Mahmoud A. Alharthi**: Conceptualization, Methodology, Software, Validation, Formal analysis, Investigation, Resources, Data Curation, Writing — Original Draft. **Rajaa M. Al‐Raddadi**: Writing — Review & Editing, Visualization, Supervision, Project administration, Funding acquisition. **Sulhi A. Alfakeh**: Conceptualization, Methodology, Validation, Investigation, Resources, Data Curation, Writing — Original Draft, Supervision. All authors have reviewed the results and approved the final version of the manuscript.

## Conflicts of Interest

The authors declare no conflicts of interest.

## Ethics Statement

This research protocol has been approved by the Directorate of Health Affairs—Jeddah, Ministry of Health with ERB number (H‐02‐J‐002).

## Consent

Informed consent was obtained from each participant or their guardian.

## Peer Review

The peer review history for this article is available at https://publons.com/publon/10.1002/brb3.70807

## Data Availability

Available upon reasonable request from the corresponding author.
